# Predicting closed quarters battle capability – Examining the influence of personality, attentional ability, 2D:4D-ratio and mindfulness on tactical performance

**DOI:** 10.1080/08995605.2024.2430578

**Published:** 2024-11-22

**Authors:** Fabio Ibrahim, Eliran Feildboy, David Nagy, Yannik Huber, Jürgen Hennig, Philipp Yorck Herzberg

**Affiliations:** a Department for Personality and Psychological Assessment, Helmut-Schmidt-University, Hamburg, Germany; b Close Quarters Battle, Project Gecko, Scheeßel, Germany; c Department for Differential and Biological Psychology, Justus-Liebig-University, Giessen, Germany

**Keywords:** Close quarters battle, resilience, big five, attention, eye-tracking

## Abstract

Close Quarters Battle (CQB) is an operational approach in confined spaces gaining increasing significance in urban combat missions. Due to its high psychophysiological demands, the CQB ability is an essential selection criterion for special forces. Until now, there has been no research on predictors of CQB capability. This study examined the influence of the Big Five personality traits, self-esteem, resilience, attentional ability, 2D:4D digit ratio, and mindfulness on the CQB performance. The German sample comprised a total of *n* = 45 individuals (*n* = 29police special forces; *n* = 16 unspecialized soldiers) who conducted psychometrics and a CQB test consisting of three scenarios. In these scenarios, two independent experts evaluated tactical behavior, weapon handling, gaze behavior, response time, and failures using a standardized behavioral observation instrument based on video recordings (external cameras and mobile eye-tracking). The results revealed that only extraversion predicted the CQB performance (β = -.40, *p* = .035). However, the mean 2D:4D ratio was strongly associated with gaze behavior (*r* = .45, *p* = .007), tactical behavior (*r* = .41, *p* = .019), and attentional ability (*p* = .57, *p* < .001). Surprisingly, the findings indicate that CQB, as a high-risk and analytical task, is better performed by introverted personnel.

**What is the public significance of this article?—** Understanding the factors influencing Close Quarters Battle (CQB) performance is vital for selecting effective special forces personnel. This study indicates that individuals with lower extraversion excel in CQB. Therefore, this implication supports optimizing the selection process and enhancing force effectiveness in urban combat.

## Introduction

In the early twenty-first century, the longest and most intense battles have occurred in cities, not in the field. The wars in Iraq, Syria, the Donbas, Libya and Yemen have all been predominantly fought not only for but, often, actually inside cities. (King, 2022, p. 69)

Urban warfare has increasingly dominated modern conflicts, with battles in Iraq, Syria, and Ukraine often occurring within cities rather than open fields (King, 2022). Over half the global population now lives in urban areas, a number projected to reach 67.2% by 2050 (Zhang et al., 2023). This urbanization, combined with the rise of asymmetric warfare, emphasizes the need for enhanced urban operational capabilities. Irregular forces benefit from the urban environment, leveraging advantages such as superior surveillance, intelligence, and subsurface operations, while limiting the strengths of regular forces (Morag, 2023).

Democratic states face the additional challenge of complying with humanitarian law, making the distinction between combatants and civilians in dense urban settings a critical and difficult task (Hills, 2004). A key tactical capability in urban warfare is Close Quarters Battle (CQB), which involves operations in confined spaces with the aim of neutralizing threats fast and minimizing risks to both combatants and civilians. Once exclusive to special forces, CQB skills are increasingly required in regular infantry due to the prevalence of urban conflicts (Shamir & Ben-Ari, 2018).

CQB demands a high level of precision, speed, and coordination, making specialized training essential (King, 2016). Given the intense cognitive, emotional, and physical demands, effective personnel selection is crucial. Research shows that cognitive abilities such as situational awareness and intuitive decision-making, alongside traits like grit, are predictors of military performance (Duckworth et al., 2007). However, direct research on predictors of CQB performance remains limited, a gap this study aims to address.

## Close Quarters Battle and Tactical Performance

CQB (synonymous with Close Quarters Combat; CQC) was developed in the 1970s by Western counterterrorist special forces in response to the terrorist attack during the 1972 Olympic Games in Munich (King, 2016). CQB entails tactical approaches in urban operational spaces at the micro-level, emphasizing corners, angles, and spatial geometry. The proximity demands the maximization of one’s force while minimizing exposure. Hyderkhan (2020) identifies surprise, speed, and violence of action as core features of CQB. In contrast to standard tactical operations, units in CQB missions must make decisions autonomously and quickly, while adapting tactically to their surroundings and the enemy’s behavior. Due to the confined space, invasive objectives, and speed, reaction time, precision, tactical flexibility, and the ability to process information are critical to success.

Beyond its impact on military operations, police special forces, particularly counterterrorist units like the American Special Weapons and Tactics Unit (SWAT), exhibit pronounced CQB expertise for apprehending armed perpetrators, counterterrorism, and hostage rescue (King, 2016). While CQB shares some similarities with urban warfare, it is distinct in its focus on immediate, tactical responses within small operational areas, rather than broader strategic military operations.

Overall, the CQB capability of both police and military remains highly relevant, despite technological developments, to meet the legal requirements of law enforcement and adherence to international humanitarian law, specifically the protection of civilians in urban conflicts.

Due to increasing relevance, the demand for personnel with CQB capabilities is growing, increasing the importance of valid assessment characteristics. The study by Kilcullen et al. (1999) showed that within the sample of existing special forces, cognitive attributes and physical fitness were not predicting special forces field performance; rather, motivation emerged as the most important performance predictor. Personality, particularly the Big Five, is commonly used in personnel selection in general and military personnel selection (Sørlie et al., 2020).

In this study, we focused on predictors that are already used in existing selection processes for special forces, such as demographic information, the Big Five personality model (Skoglund et al., 2020), self-esteem, and attentional performance (Heusler & Sutter, 2022). We also examined resilience and mindfulness, as both constructs are gaining increasing attention in high-risk occupations (Nassif et al., 2023; Johnson et al., 2014). Lastly, we included the 2D:4D ratio as an indicator of prenatal testosterone concentration as a predictor, given its possible relevance in high-risk occupation selection (Kozieł et al., 2018). Therefore, insights regarding the association between variables used in special forces selection, such as the Big Five and CQB performance, can be leveraged by military and police psychologists with minimal effort.

## Personality as a Possible Predictor for CQB Performance

Personality, commonly assessed through the Big Five traits, plays a critical role in determining person-job fit, especially in high-risk professions like the military (Sørlie et al., 2020). Research shows that military recruits tend to score lower on agreeableness, neuroticism, and openness compared to civilians (Jackson et al., 2012). Special forces personnel, in particular, exhibit even lower levels of extraversion, agreeableness, and neuroticism, distinguishing them from the broader military population (Skoglund et al., 2020).

Extraversion, characterized by social engagement and high activity levels, has been found to be negatively associated with military training performance due to its link to lower risk-aversity and lower risk perception (Hartmann et al., 2003; Ishfaq et al., 2020; Oehler & Wedlich, 2018). Persuasiveness, motivating others, supervision, and dominance (Russell et al., 1996) are associated with general special forces job performance and extraversion could function as a predictor of those interpersonal skills. In CQB contexts, where precision and calculated decisions are crucial, extraversion may hinder performance as extraverts tend to react quickly, often at the expense of accuracy (Patterson et al., 1987).

Neuroticism, associated with emotional instability and anxiety, has been shown to negatively impact performance in military training environments, particularly in high-stress roles like aviation (Campbell et al., 2009a). Neurotic individuals are more sensitive to stress, exhibit stronger physiological reactions to threats, and are less resilient under pressure (Liu et al., 2012; Norris et al., 2007), which may impair CQB performance.

Openness, linked to curiosity and complexity, is typically lower in special forces personnel and has been found to negatively affect marksmanship performance (Buskerud et al., 2022). This may reflect a preference in special forces for individuals who are more pragmatic and less open to novel or unconventional ideas (Huijzer et al., 2022).

Conscientiousness, a predictor of long-term goal attainment and self-discipline, positively correlates with success in structured environments like military academies (Bobdey et al., 2021; Piedmont et al., 1999) and with performance across a broad range of domains (Zell & Lesick, 2022). Specifically, the achievement motivation facet of conscientiousness has been shown to predict special forces performance as evaluated by supervisors (Kilcullen et al., 1999). While conscientiousness is crucial for long-term skill development, it may be less immediately relevant to CQB tasks that require split-second decision-making (Roberts et al., 2018).

Agreeableness, reflecting a prosocial orientation, has been found to be lower in military personnel, particularly in pilots and soldiers (Campbell et al., 2009b; Klee & Renner, 2016). However, it may positively influence group dynamics and synergy in CQB squads, where teamwork is essential for mission success (Bartone et al., 2002; Halfhill et al., 2005).

### Self-Esteem

In addition to the Big Five, self-esteem is a central personality trait defined as a dispositional assessment of one’s worth (Rosenberg, 1965) and an indicator of confidence in one’s actions. Previous research has established self-esteem as a robust predictor of self-confidence (Coudevylle et al., 2011), with self-confidence, defined as a sense of assurance (Compte & Postlewaite, 2004), generally being positively linked to athletic performance, particularly in high-pressure contexts (Navia et al., 2019;). Under pressure, athletes tend to focus more on task-irrelevant cues, reducing relevant information processing and performance (Oudejans et al., 2011). Self-esteem acts as a buffer and improves coping with pressure (Vuillemin et al., 2018) and is therefore expected to predict a higher CQB-perfromance.

### Resilience

Resilience, defined as the ability to overcome challenges and recover from adversity (Britt et al., 2013), is predicted by conscientiousness and extraversion (Iversen et al., 2023). It correlates with higher self-esteem following setbacks (Oshio et al., 2003) and enhanced athletic performance (Hosseini & Besharat, 2010). In high-risk occupations, where ambiguous environments increase setbacks, both resilience and physical fitness are crucial due to the intense physical demands during operations and training (Picano et al., 2022). Additionally, mindfulness interventions are increasingly recognized for improving resilience, focus, and reducing anxiety in operational occupations (Greene & Staal, 2017; Johnson et al., 2014).

### Mindfulness

Mindfulness describes the ability to focus on the current moment, to shift attention to new stimuli, and process stimuli without reactivity or judgment (Dane, 2011). The meta-analysis by Mesmer-Magnus et al. (2017) indicates that a dispositional consideration of mindfulness (trait mindfulness) is positively associated with self-confidence, emotion regulation, and job performance and negatively associated with perceived stress and anxiety. Mindfulness training, such as Mindfulness-Based Attention Training, enhances marksmanship performance under physical stress, attentional performance, and emotion regulation (Nassif et al., 2023). A nonjudgmental attitude, characterized by focusing on the present moment without judgment (Marlatt and Kristeller, 1999), is associated with reduced anxiety, rumination, and worry (Barcaccia et al., 2019). Consequently, higher levels of dispositional mindfulness and nonjudgment may positively predict CQB performance.

## Finger Digit Ratio − 2D:4D

The 2D:4D ratio describes the relationship between the length of the index and ring fingers and relates to prenatal testosterone concentrations, with a lower 2D:4D ratio indicating a higher prenatal testosterone-estrogen ratio (Manning et al., 1998). Females generally have a higher 2D:4D ratio than males (Manning et al., 2014) and a higher ratio is linked to uncertainty avoidance and neuroticism (Manning & Fink, 2011). Moreover, a lower ratio is associated with aggression (Kilduff et al., 2013), mental toughness (Zamani Sani et al., 2022), sensation-seeking (Fink et al., 2006), athletic performance (Hönekopp & Watson, 2010), suggesting a potential association with CQB performance.

## Attention

Another psychological predisposition associated with athletic performance is the attentional capacity (Zwierko et al., 2014). Attention involves selecting immediately perceived relevant external stimuli and selective attention describes the rapid discrimination between target stimuli and distractors while filtering out irrelevant information (Allport, 1987). The ability to selectively process information is a fundamental aspect of goal-directed actions and the executive functions (Blair, 2017). Therefore, attentional training has shown positive effects, such as in live-fire scenarios (Heusler & Sutter, 2022) and, we anticipate a positive influence of the attentional ability on the CQB performance.

### The Current Study

Due to CQB’s increasing relevance and high difficulty, appropriate personnel selection is crucial to enhance the likelihood of training and mission success. To the best of our knowledge, there is no research exploring predictors of CQB performance. Therefore, this study aims to identify which dispositions and personality traits are associated with CQB performance and suitable as selection criteria. We hypothesize:

H1: Extraversion is negatively related to the CQB performance.

H2: Neuroticism is negatively related to the CQB performance.

H3: Openness is negatively related to the CQB performance.

H4: Self-esteem is positively related to the CQB performance.

H5: Resilience is positively related to the CQB performance.

H6: Nonjudging is positively related to the CQB performance.

Further, a low 2D:4D ratio is associated with athletic performance and the attentional ability is associated with military performance. Therefore, we assume:

H7: The 2D:4D ratio is negatively related to the CQB performance.

H8: The attentional ability is positively related to the CQB performance.

## Method

### Design and Procedure

This experimental study on CQB performance was conducted between October and November 2023, with voluntary participation. Ethical approval was obtained from the Helmut-Schmidt-University (Ethics approval number: 2023_010), and all participants provided informed consent for anonymized data processing and were informed of their right to withdraw at any time.

The investigation began with an online survey, collecting demographic, biographical, and personality data. Participants used pseudonymous subject codes to link survey responses with performance variables. On examination day, participants completed the following tasks: (1) an attention test (d2-R; Brickenkamp et al., 2010), (2) sleep quality assessment as a control variable (duration, waking frequency, recovery feeling), (3) 2D:4D ratio measurement, and (4) a 30-minute relaxation phase (ear protection, sleep mask) to standardize arousal levels. Participants then equipped chest straps for heart rate monitoring (Polar H10) and prepared their gear (helmet, protective vest) for the CQB scenarios. Before the CQB scenarios, participants were briefed, equipment was checked, and helmet cameras (GoPro Hero 10) and eye-tracking devices (Viewpoint System 19) were attached. The CQB pretest included three room-clearing scenarios where participants engaged simulated threats (red light and target) or responded to real individuals based on threat level. Police special forces used Walther P99 FX training ammunition, while soldiers used airsoft Glock 17s.Psychophysiological measures including heart rate, saliva cortisol, and alpha-amylase are reported in Ibrahim et al. (2024).

### Sample

The total sample comprised *N* = 45 individuals (*n* = 3 female; *M* = 31.27; *SD* = 6.95). Recruitment took place at the Helmut-Schmidt-University/University of the German Armed Forces in Hamburg and through a cross-state inquiry of police special forces units. The overall sample included *n* = 29 police special forces, with *n* = 12 from the Special Task Force (German: Spezialeinsatzkommando; SEK), *n* = 17 from the Mobile Task Force (German: Mobiles Einsatzkommando; MEK) from various federal states, and *n* = 16 unspecialized soldiers. The police special forces examined here are officers who have undergone several months of specialized training under high physical and psychological stress to prepare for special operations such as hostage situations or counter-terrorism. CQB, in particular, forms a core component of tactical training in the basic training and mission preparation of police special forces. They do not perform regular police duties but are deployed in particularly high-risk situations. The predominant educational attainment was a Bachelor’s degree, constituting *n* = 20 (44.44%), and participants reported an average of *M* = 9.98 (SD = 6.60) years of service. The mean CQB experience was *M* = 77.47 hours (*SD* = 106.08; range = 400). Demographic details are displayed in the supplements (Tab. A1). Assuming a medium correlational effect size (*ρ* = 0.50), an alpha level of .05, and a power of .80, the minimum required sample size is *n* = 30. The data is accessible online at: https://osf.io/n4ugp/?view_only=21b8773f058a42039ec0a0fd9cef203e.

### Close Quarters Battle Performance Test

The CQB paradigm for measuring the CQB performance consisted of three standardized scenarios set in distinct spatial environments. Prior to each scenario, participants were intentionally kept unaware of the room configuration or situational details. The first scenario presented a confined space with numerous angles and doors (restroom cabins), featuring no hostile contact. The second scenario involved a larger and more expansive environment. In this setting, the operator had to engage a red-marked target (shooting) before verbally subduing a suddenly appearing civilian holding an object (requiring speed, precision, discrimination, and inhibition). The third scenario confronted the operator with an armed adversary upon opening the door, who, instead of being engaged, needed to be subdued verbally (emphasizing discrimination and inhibition).

### Measures

#### CQB Performance

Two independent experts (soldiers with specialized tactical training and experience, who collaborated with the training and development departments of police special forces and military personnel to formulate the evaluation criteria) assessed CQB performance using multi-perspective videos (head-mounted cameras, mobile eye-tracking, external cameras). A standardized rating instrument was developed by independent CQB experts with a police or military background, consisting of 9 items forming five scales. The items served as behavioral anchors, and assessors rated them on a scale ranging from 1 (*does not apply at all*) to 7 (*applies completely*).

The first scale, *tactical behavior*, included identifying threat points, angle coverage, and withdrawal from the danger zone. Scale 2, *weapon handling*, comprised of loading activities, weapon and gaze direction, and trigger behavior. Scale 3, *gaze behavior*, included target perception, initial fixation, and search efficiency. A fixation was considered valid if it lasted for a minimum of 200 ms (Niehorster et al., 2020), and the sequence of fixations and fixation on critical points were assessed. Scale 4, *response time*, involved evaluating the time elapsed between target identification and response. The fifth and final scale, mistakes, encompassed the accumulation of errors (e.g., shooting civilians), with 7 points deducted from the total score for each mistake. The overall CQB performance scale constituted the sum of the three scenario scores. Interrater reliability for the assessment of CQB performance was evaluated using a two-way mixed effects model ICC (Gisev et al., 2013). The ICC results demonstrated substantial agreement and very good reliability across the five performance scales (ICC = .834, *p* < .001).

#### The Big Five Inventory

The Big Five Inventory (BFI; Rammstedt & Danner, 2017) comprises 45 items across five scales, measured on a 5-point Likert scale from 1 (strongly disagree) to 5 (strongly agree). The reliability of the scales is good to very good, with α = .78–.83.

#### Rosenberg Self-Esteem Scale

The German version of the Rosenberg Self-Esteem Scale (RSES; Rosenberg, 1965) was employed in the adaptation by Von Collani and Herzberg (2003). The instrument consists of ten items, answered on a 4-point Likert scale, ranging from 1 (*not true at all*) to 4 (*entirely true*). The internal consistency is very good, with α = .84.

#### Resilience Scale

The Resilience Scale 11 (RS-11; Schumacher et al., 2005) is a brief version designed to assess psychological resilience. It comprises 11 items, measuring the extent on a 7-point Likert scale ranging from 1 (*I do not agree*) to 7 (*I agree*). The internal consistency is excellent, with α = .91.

#### Five Facet Mindfulness Questionnaire

The German version of the Five Facet Mindfulness Questionnaire (FFMQ-G; Michalak et al., 2016) comprises a total of 39 items, rated on a 5-point Likert scale from 1 (*never or very rarely true*) to 5 (*very often true*). The five scales – observing, describing, acting with awareness, nonjudging, and nonreactivity – contribute to an FFMQ total score. The internal consistency ranges from acceptable to very good (α = .74–.90).

#### Finger Digit Ratio − 2D:4D

The computer-assisted measurement of the index finger (2D) and ring finger (4D) involved scanning the left and right hand (HP Color Laser 179fwg) and graphically determining the finger length. The ratio served as an indicator of prenatal testosterone concentration. The digital images of both hands were analyzed using the browser-based software ImageJ (https://ij.imjoy.io/) in order to measure the length of the 2^nd^ and 4^th^ digit of each hand. Afterwards the ratio was computed (length of 2D divided by length of 4D).

#### Attention Test – d2-R

The d2-R attention test (Brickenkamp et al., 2010) is a paper-pencil test designed to assess attention performance based on speed and precision in marking targets (d’s with two dashes). The total duration of the procedure is 4 minutes and 40 seconds (14 lines at 20 seconds each). The concentration performance score as the difference between the number of hits and marked distractors demonstrates very good reliability (test-retest reliability = .77–.95).

### Statistical Analysis

We employed the R software (R Core Team, 2022) for statistical analysis, utilizing independent t-tests to assess group differences. Hypotheses 1–6 were evaluated using a hierarchical regression model (stepwise), with the predictors being the Big Five, self-esteem, resilience, and nonjudging, and CQB performance serving as the dependent variable. Additionally, the CQB facets – tactical behavior, weapon handling, gaze behavior, and response time – were investigated using four separate hierarchical regressions. For Hypotheses 7–8, Pearson product-moment correlations were utilized, with Spearman’s correlation employed when parametric testing assumptions were not met. Effect size interpretation followed the guidelines set forth by Gignac and Szodorai (2016); *r* = .10/.20/.30 for small/moderate/large effects). Outlier analysis utilized Mahalanobi’s distance measure and individuals with a d-squared value of *p* < .001 were visually inspected and excluded in case of doubts about plausibility.

## Results

The multivariate outlier analysis revealed no jumps in d-squared values (*p* < .001), and the visual data inspection did not identify any outliers. Consequently, all datasets were included in the subsequent analysis.

### Comparison of Subsamples

The special forces displayed a higher age, more years in service, and more experience in CQB than non-specialized soldiers. However, regarding personality traits and mindfulness, no differences were observed between the groups (see Table S1A). The comparison of CQB performance between the groups demonstrated, as expected, higher scores for the Special Forces in tactical behavior (*t*(31) = −5.508, *p* < .001, *d* = 1.993), weapon handling (*t*(32) = −4.639, *p* < .001, *d* = −1.665), gaze behavior (*t*(32) = −3.064, *p* = .004, *d* = −1.099), and overall CQB performance (*t*(36) = −5.352, *p* < .001, *d* = −1.830). In terms of response time (*t*(32) = −1.456, *p* = .155, *d* = 0.522) and mistakes (*t*(32) = −1.801, *p* = .081, *d* = −0.646), no statistically significant differences were found (Table 1).

**Table 1.** CQB performance between non-specialized and special forces.

	*t*	*df*	*P*	Cohen´s *d*	95% CI	
					Lower	Upper
Tactical behavior	−5.508	31	<.001	−1.993	−2.847	−1.118
Weapon handling	−4.639	32	<.001	−1.665	−2.467	−0.843
Gaze behavior	−3.064	32	0.004	−1.099	−1.845	−0.339
Respone time	−1.456	32	0.155	−0.522	−1.233	0.196
Mistakes	−1.801	32	0.081	−0.646	−1.362	0.079
CQB total performance	−5.352	36	<.001	−1.830	−2.612	−1.029

95% CI for for Cohen´s *d*.

### Personality as a Predictor of CQB Performance

Five hierarchical regressions were computed to examine the relation of self-reported personality with CQB total performance and its facets (Table 2). The results indicate that the CQB total performance was negatively predicted by extraversion (β = −.403, *p* = .035), supporting H1. Neuroticism and openness did not predict CQB total performance, providing no support for H2 and H3. Similarly, the personality traits self-esteem, resilience, and nonjudging showed no association with CQB, failing to support H4 – H6. Furthermore, examining the CQB facets revealed that extraversion negatively predicted tactical performance (β = −.452, *p* = .028) and weapon handling (β = −.482, *p* = .011). However, gaze behavior and reaction time exhibited no correlation with the investigated personality variables.

**Table 2.** Hierarchical regression analysis predicting CQB total performance and performance facets.

Model	Predictor	Beta	*t*	*p*	*F*	R	R^2^	Adj. R^2^
M1: CQB total performance				.390	1.090	.417	.174	.014
	Extraversion	−.403	−2.210	.035				
	Neuroticism	.046	0.204	.839				
	Openness	−.115	−0.665	.511				
	Self-esteem	−.141	−0.599	.553				
	Resilience	.233	0.905	.372				
	Nonjudging	.228	0.998	.326				
M2: Tactical performance				.310	1.259	.474	.225	.046
	Extraversion	−.452	−2.335	.028				
	Neuroticism	.087	0.379	.708				
	Openness	−.147	−0.802	.430				
	Self-esteem	−.121	−0.477	.637				
	Resilience	.271	1.022	.316				
	Nonjudging	.214	0.906	.373				
M3: Weapon handling				.078	2.166	.570	.325	.175
	Extraversion	−.482	−2.732	.011				
	Neuroticism	−.275	−1.300	.205				
	Openness	−.166	−0.988	.332				
	Self-esteem	.209	0.909	.371				
	Resilience	.088	0.363	.720				
	Nonjudging	−.026	−0.119	.906				
M4: Gaze behavior				.671	0.675	.361	.130	−.063
	Extraversion	−.274	−1.307	.202				
	Neuroticism	.048	0.201	.842				
	Openness	−.161	−0.844	.406				
	Self-esteem	.004	0.017	.987				
	Resilience	−.018	−0.065	.948				
	Nonjudging	−.023	−0.094	.926				
M5: Response time				.239	1.432	.491	.241	.073
	Extraversion	.162	0.866	.394				
	Neuroticism	.286	1.277	.212				
	Openness	−.265	−1485	.149				
	Self-esteem	−.062	−0.253	.802				
	Resilience	−.204	−0.789	.437				
	Nonjudging of internal experience	.066	0.288	.775				

*N* = 34 (25 police special forces; 13 unspecialized soldiers).

### Psycho-Physiological Dispositions as Predictor of Performance

Investigating the psycho-physiological dispositions revealed that the 2D:4D ratio, serving as an indicator of prenatal testosterone concentration, exhibited no association with the CQB performance (*r* = .271, *p* = .100; H7). However, examining the CQB facets, a strong association between the mean 2D:4D ratio and tactical behavior (*r* = .405, *p* = .019; Tab. 2A) and gaze behavior (*r* = .451, *p* = .007) were found. Further, the 2D:4D ratio and attentional ability (*p* = .569, *p* < .001) exhibited a strong relationship. Nevertheless, attentional ability displayed no correlation with CQB performance (*p* = .108, *p* = .517), leading to the rejection of H8.

## Discussion

This study explored predictors of CQB performance, aiming to identify key characteristics for selecting personnel in specialized and special forces roles. Results showed that police special forces outperformed non-specialized soldiers in tactical behavior, weapon handling, gaze behavior, and overall CQB performance. These differences can largely be attributed to the specialized training and operational experience of police forces. However, the lack of significant differences in response time suggests that this may be a dispositional trait less influenced by training, highlighting its importance in personnel selection.

Extraversion was the only Big Five trait negatively associated with CQB performance, particularly in tactical behavior and weapon handling. Extraverted individuals are more prone to risk-taking and lower risk perception (Ishfaq et al., 2020; Oehler & Wedlich, 2018), which may hinder performance in CQB scenarios where precision and caution are crucial. This finding aligns with Skoglund et al. (2020), who reported lower extraversion levels in special forces compared to regular soldiers.

Interestingly, neuroticism and openness, typically considered important in military performance (Fornette et al., 2023; Huijzer et al., 2022), were not significant predictors in this study. This could be due to the pre-selected, homogenous sample, where participants likely exhibited lower levels of neuroticism, reducing variability in this trait. This explanation parallels findings from Kilcullen et al. (1999), who reported a lack of physical fitness and cognitive ability effects in pre-selected special forces samples.

Similarly, resilience, self-esteem, and the mindfulness facet nonjudging did not predict CQB performance. The absence of associations might stem from the study environment not generating enough pressure, or the sample’s high baseline resilience (Sudom et al., 2014). Mindfulness training, though beneficial in high-stress military settings (Nassif et al., 2023), may not directly influence performance in simulated CQB scenarios.

The investigation of the 2D:4D ratio as an indicator of prenatal testosterone showed no significant correlation with overall CQB performance, though strong correlations were found with tactical and gaze behavior. This is consistent with research suggesting that expert performance, particularly in gaze patterns, is associated with systematic and efficient attention allocation (Papesh et al., 2021). The correlation between the 2D:4D ratio and attentional ability suggests this measure could be a dispositional factor in information processing, making it a promising area for future research.

Moving forward, it would be beneficial to include additional predictors such as cognitive abilities and intelligence, which have proven relevant to military performance (Calleja et al., 2020), as well as decision-making skills, particularly naturalistic-intuitive decision-making, which is critical in operational contexts (Männiste et al., 2019). Additionally, physical fitness, already established as a key predictor of military performance (Silva et al., 2020), should be further explored in relation to CQB.

### Limitations

This study has several limitations that should be addressed in future research. A primary limitation is the small sample size, which restricted the ability to detect moderate effect sizes. This is evident in the regression models, none of which reached statistical significance. Future studies should increase sample sizes to improve statistical power and detect subtler predictors of personnel performance. Additionally, the sample included both soldiers and police special forces, two high-risk occupational groups with distinct socialization processes and demographic differences. The police special forces were older and had more CQB experience, leading to potential unsystematic variance. Future studies should aim for more homogeneous samples in terms of demographics to improve comparability.

Another limitation is the reliance on self-report measures, which may be biased by social desirability, particularly in military contexts (Thunholm, 2001). Future research could incorporate external or behavior-based personality assessments to reduce this bias. Russell et al. (2023) highlighted that motivational and personality characteristics are primary drivers of contextual performance. Consequently, an important direction for future research could be to examine contextual performance indicators, such as supportive and social behaviors, as personality-performance validities may increase when contextual performance is measured separately (Borman & Motowidlo, 1997).

For measuring the 2D:4D ratio, it is recommended that two independent raters assess this variable to ensure accuracy and calculate inter-rater reliability. Additionally, this study did not measure shooting precision, which could be a valuable extension of CQB performance metrics (Ibrahim et al., 2023). Furthermore, only individual CQB scenarios were analyzed, whereas real-world operations typically involve teams. Therefore, future research should explore how individual predictors and the interaction of personality traits affect group CQB performance.

### Practical Implications

This study, with its high ecological validity through real-life environments and standardized scenarios, reveals that lower extraversion is linked to higher CQB ability in soldiers and police officers. These findings are crucial for specialized force selection and aid military psychologists in interpreting personality assessments and developing job profiles. The 2D:4D ratio’s association with tactical behavior, gaze behavior, and attentional ability suggests its potential as a valuable variable in roles demanding high attention, like air traffic controllers or snipers. Future research should validate these findings with larger samples and further explore CQB’s stress responses, training effects, and team dynamics to refine personnel selection for CQB operations.

## Supplementary Material

Open_Science_Badges_Disclosure_Form_Annotated_FI.docx

## Data Availability

All data is openly available in the Open Science Framework under https://osf.io/n4ugp/?view_only=21b8773f058a42039ec0a0fd9cef203e.
